# Anti-GD2/4-1BB chimeric antigen receptor T cell therapy for the treatment of Chinese melanoma patients

**DOI:** 10.1186/s13045-017-0548-2

**Published:** 2018-01-03

**Authors:** Jiayi Yu, Xiaowen Wu, Junya Yan, Huan Yu, Longwen Xu, Zhihong Chi, Xinan Sheng, Lu Si, Chuanliang Cui, Jie Dai, Meng Ma, Tianxiao Xu, Yan Kong, Jun Guo

**Affiliations:** Department of Renal Cancer and Melanoma, Peking University Cancer Hospital & Institute, Collaborative Innovation Center for Cancer Medicine, Key Laboratory of Carcinogenesis and Translational Research (Ministry of Education/Beijing), Beijing, 100142 China

**Keywords:** CAR-T, GD2, 4-1BB, Melanoma, Immunotherapy

## Abstract

**Background:**

Chimeric antigen receptor (CAR)-engineered T cells have demonstrated promising clinical efficacy in patients with B cell lymphoma. However, the application of CAR-T cell therapy in the treatment of other solid tumors has been limited. We incorporated 4-1BB into the anti-GD2 CAR-T cells to test their cytotoxicity in melanoma in vitro and in vivo. Moreover, we reported the expression of ganglioside GD2 in non-Caucasian melanoma populations for the first time, thus providing a basis for future clinical research.

**Methods:**

This study included tumor samples from 288 melanoma patients at the Peking University Cancer Hospital & Institute. Clinical data were collected. Immunohistochemical assays using antibodies against ganglioside GD2 were performed on formalin-fixed, paraffin-embedded specimens. The ability of ganglioside GD2 CAR-T cells to kill ganglioside GD2^+^ melanoma cells was evaluated in vitro and in a patient-derived xenograft (PDX) model.

**Results:**

Among the 288 samples, 49.3% of cases (142/288) demonstrated positive staining with ganglioside GD2. The median survival time in patients exhibiting ganglioside GD2 expression was significantly shorter than that in patients without ganglioside GD2 expression (31 vs. 47.1 months, *P* < 0.001). In the present study, CAR was constructed using a GD2-specific scFv (14.G2a), T cell receptor CD3ζ chain, and the CD137 (4-1BB) costimulatory motif. In addition, the GD2.BBζ CAR-T cells demonstrated specific lysis of ganglioside GD2-expressing melanoma cells in vitro. In two PDX models, mice that received intravenous or local intratumor injections of GD2.BBζ CAR-T cells experienced rapid tumor regression.

**Conclusions:**

These data demonstrate that the rate of GD2 expression in Chinese patients is 49.3%. GD2.BBζ CAR-T cells can both efficiently lyse melanoma in a GD2-specific manner and release Th1 cytokines in an antigen-dependent manner in vitro and in vivo. Anti-GD2/4-1BB CAR-T cells represent a clinically appealing treatment strategy for Chinese melanoma patients exhibiting GD2 expression and provide a basis for future studies of the clinical application of immunotherapy for melanoma.

**Electronic supplementary material:**

The online version of this article (10.1186/s13045-017-0548-2) contains supplementary material, which is available to authorized users.

## Background

Chimeric antigen receptor (CAR)-engineered T cells have demonstrated significant promising clinical efficacy in patients with hematologic malignancies [1, 2]. The complete response (CR) rate of CD19-specific CAR-T clinical trials ranges from 50 to 90% [2, 3]. The success of this therapy relies on the genetic addition of synthetic CARs to T cells, which enable them to target tumor cells in a major histocompatibility complex (MHC)-unrestricted manner. Despite recent successes in this field, the application of CAR-T cell therapy for the treatment of other solid tumors has remained challenging, largely due to the lack of appropriate tumor-specific antigens [4, 5] and insufficient localization and persistence of CAR-T cells [6, 7]. Thus, the identification of precise tumor-specific antigens and the appropriate construction and design of CARs are vital for this immunotherapy.

GD2 gangliosides are sialic acid-containing glycosphingolipids that play a role in signal transduction, cell-cell recognition, cell proliferation, cell migration, and tumor cell metastasis [8, 9]. Ganglioside GD2 is overexpressed on several solid tumors, including melanoma, neuroblastoma, Ewing’s sarcoma, and even some mesenchymal stem cells [10, 11]. Due to its high level of expression in tumors and restricted expression in normal tissues, GD2 is a good target for cancer therapy [12, 13]. Based on its immunogenicity, therapeutic functions, oncogenicity, and other factors, GD2 is a very attractive target and was ranked 12th among the most important cancer antigens by the National Cancer Institute pilot program [14]. Anti-GD2 monoclonal antibodies were approved by the Food Drug and Administration (FDA) for the treatment of neuroblastoma in March 2017 [15]. In a phase I study, the murine IgG3 monoclonal antibody (MoAb) 3F8, which specifically targets ganglioside GD2, was intravenously administered to patients with neuroblastoma or melanoma. Antitumor responses occurred in 7 out of 17 patients, which ranged from complete clinical remissions to mixed responses, and the response rate of the melanoma was 4/9 [16]. Moreover, ganglioside GD2 chimeric antigen receptor T cells have already been studied in patients with neuroblastoma in a phase I clinical trial, which indicated that anti-GD2 CAR-T cells are safe and mediate modest antitumor activity [17].

Previous studies have shown that anti-GD2 CAR-T cells in which the CD28 and OX40 endodomains have been incorporated exhibit antitumor activity on melanoma in vitro and in vivo [18]. However, research has demonstrated that CD28 co-stimulation could augment T cell exhaustion, which is a major factor limiting antitumor responses in the stimulation of chronic antigens [19–21], whereas 4-1BB co-stimulation could ameliorate this situation [22]. The secretion of cytokines and the proliferative ability of exhausted T cells are decreased as apoptosis, and immune-related inhibitory receptors are increased [23]. Moreover, it has been confirmed that early T cell exhaustion is the primary factor limiting the cytotoxic activity of CAR-T cells [22].

Hence, we generated GD2 CAR-T cells incorporated with 4-1BB to test their cytotoxic activity in melanoma in vitro and in vivo. Moreover, we reported the expression levels of ganglioside GD2 in non-Caucasian melanoma populations for the first time, which provided a basis for future clinical research.

## Methods

### Patients and tissue samples

This study included lesion samples from 288 melanoma patients who visited the Peking University Cancer Hospital between November 2009 and November 2016. Written informed consent was obtained from all patients. All diagnoses of melanoma were confirmed histopathologically. Clinical data, including age, sex, American joint committee on cancer (AJCC) M stage, thickness, ulceration, metastasis, and overall survival (OS, follow-up persisted until March 2017, lost to follow-up or death), were collected. This study was approved by the Medical Ethics Committee of the Beijing Cancer Hospital & Institute and was conducted in accordance with the Declaration of Helsinki. The datasets used and/or analyzed during the current study are available from the corresponding author upon request.

### Immunohistochemistry

Formalin-fixed, paraffin-embedded (FFPE) tissue sections were examined by immunohistochemistry (IHC) using the monoclonal mouse anti-human ganglioside GD2 antibody 14.G2a (Santa Cruz, sc-53831). A standard Strept-avidin horseradish immunoperoxidase method was used for human Ganglioside GD2 staining. Primary antibodies were diluted in buffer containing 10% normal goat serum. The tissue sections were deparaffinized with Xylene for 30 min and rehydrated in decreasing concentrations of ethanol. Endogenous peroxidases were blocked with 30% H_2_O_2_ diluted in phosphate-buffered saline (PBS) for 15 min. For antigen retrieval, slides were heated in a pressure cooker in EDTA (pH 8.5) for 2 min and 30 s, followed by cooling to room temperature (RT) in the same buffer. For antigen blocking, the slides were blocked with normal goat serum with 1 h. After washing, slides were incubated with the primary antibody overnight at 4 °C (dilution 1:25). Three 5-min washes in buffer were conducted after each incubation. The slides were then incubated with the secondary antibody, anti-rabbit/mouse antibody (DAKO) (30 min at RT), followed by staining with AEC for 5-30 min at RT until coloration was achieved, counterstaining with hematoxylin followed by staining, and sealing with water-soluble encapsulating agent. Staining intensity and percentage were independently scored by three pathologists as 0, 1, or 2 (“0” as negative, and “1” and “2” as positive).

### Cell lines and primary cell culture

The 293T, SK-MEL-5 (catalog no. HTB-70), and WM-266-4 (catalog no. CRL-1676) cell lines were obtained from the American Type Culture Collection (ATCC). A875 (catalog no. ZY-H405) melanoma cell lines were purchased from Zeye Biotechnology Company (Shanghai, China). HMV-II melanoma cell lines were a gift from Dr. Xu (Abramson Cancer Center of the University of Pennsylvania, Philadelphia, PA). The mucosal melanoma GAK cell lines (catalog no. JCRB0180) was purchased from the Japanese Collection of Research Bioresources Cell Bank (JCRB). The 293T, A875, SK-MEL-5, and WM-266-4 cells were maintained in Dulbecco’s Modified Eagle Medium (DMEM, Invitrogen) supplemented with 10% FBS, 100 UI/ml penicillin, and 100 μg/ml streptomycin. HMV-II cells were maintained in F-10 (Invitrogen), and GAK cells were maintained in F-12 (Invitrogen) supplemented with 10% FBS, 100 UI/ml penicillin, and 100 μg/ml streptomycin.

The MMYC-3 (mucosal primary melanoma cell), MMYC-7 (mucosal primary melanoma cell), and AMYC-5 (acral primary melanoma cell) cell lines were derived from a patient-derived xenograft (PDX) model. The tumor tissue was minced into 1-mm^3^ fragments and resuspended in 30 ml of DMEM containing 50× collagenase IV (Invitrogen) and 1× DNase (Takara, Kusatsu, Japan). After a 2-h incubation at 37 °C, the suspensions were collected and slowly transferred onto a 15-ml Histopaque (Sigma, St. Louis, MO), and then, the interface cell fraction was collected after centrifugation. The cells were then maintained in serum-free stem cell medium supplemented with growth factors at 37 °C in 5% CO_2_.

### Construction of the anti-GD2 CAR

The chimeric GD2/CAR is composed of GD2 scFv and a 4-1BB-CD3ζ expression cassette that was designed and synthesized by the GeneChem Biotechnology Company (Shanghai, China), as shown in Fig. 3a. The GD2 scFv was derived from a high-affinity 14.G2a monoclonal antibody. The 4-1BB-CD3ζ expression cassette contains the hinge and transmembrane (TM) region of CD8α. GD2 scFv and 4-1BB-CD3ζ were connected in-frame by overlap PCR. The generated GD2/CAR was verified by DNA sequencing and cloned into the BamHI sites of a lentiviral vector (Genechem Biotechnology, China); the resultant product was named GD2.BBζ CAR. The specific structure of the viral vector is shown in Additional file 1: Figure S1. The intracellular domain of the CARs has the self-cleaving 2A peptide connected to an EGFP green fluorescent label. The sequences of all PCR primers are available upon request.

### Transduction of lentiviral GD2/CAR

After informed consent was obtained from normal volunteers, peripheral blood mononuclear cells (PBMCs) were isolated by Ficoll-Paque PLUS. T cells were transfected with an Easy-T kit from GeneChem. Briefly, isolated T cells/PBMCs were activated on a plate precoated with S buffer (EASY-T cell infection activation kit, catalog no. LCR6018, GeneChem) at a concentration of 0.5 × 10^6^ cells/ml in complete TexMACS media (Miltenyi) supplemented with 5% human serum and 300 IU IL-2 (Mitenyi). Two days later, the stimulated T cells were washed and resuspended at 0.5 × 10^6^ cells/mL with Trans B buffer (EASY-T cell infection activation kit, catalog no. LCR6018, GeneChem). CAR-encoding lentivirus (GD2.BBζ CAR) was thawed and added into the cells (virus titer: 2 × 108TU/ml, MOI = 3). The cells were seeded onto plates that had been coated for 2 h with Trans A buffer (EASY-T cell infection activation kit, catalog no. LCR6018, GeneChem). Then, the transduced T cells were cultured at 37 °C and 5% CO_2_ and expanded to maintain a cell concentration of 0.5–1 × 106 cells/ml.

### Flow cytometry

FITC-, PE-, or perCP-conjugated anti-CD3, CD4, CD8, CD25, PD-1, TIM-3, LAG-3 monoclonal antibodies, and PE Annexin V apoptosis detection kit were used to stain lymphocytes (all from BD Bioscience), whereas the anti-GD2 mAb (Santa Cruz) was used to label melanoma cells. A GD2 isotype antibody (Santa Cruz) was used as a negative control for the detection of GD2 expression. The proliferation of GD2.BBζ CAR and non-transduced T cells in the presence of tumor cells was evaluated by fluorescence-activated cell sorting (FACS) analysis after labeling the T cells with using the CellTrace™ Far Red Cell Proliferation Kit (Invitrogen).

### Cytotoxicity assays

The cytotoxic activity of the GD2.BBζ CAR and non-transduced CAR-transduced T cells was evaluated using the CytoTox 96® Non-Radioactive Cytotoxicity Assay (Promega). We evaluated lactate dehydrogenase (LDH) release at 4 and 24 h in culture with effector-to-target (E:T) ratios of 40:1, 20:1, 10:1, and 5:1.

### Co-culture experiments

GD2.BBζ CAR and non-transduced T cells were plated at 1 × 10^6^ cells per well on a 96-well plate at a 20:1 ratio with 293T, SK-MEL-5, HMV-II, and GAK cells. Interleukin-2 (IL-2), interleukin-4 (IL-4), interleukin-5 (IL-5), interleukin-10 (IL-10), tumor necrosis factor (TNF-α), and interferon-γ (IFN-γ) cytokine release after 24 h of culture was measured using the cytometric bead array (CBA) human Th1/Th2 cytokine kit (BD Bioscience).

GD2.BBζ CAR and non-transduced T cells labeled by CellTrace™ Far Red were plated at 5 × 105 cells per well on a 12-well plate at a 20:1 ratio with 293 T, WM-266-4, HMV-II, and GAK cells, and the percent of GD2.BBζ CAR and non-transduced T cells was evaluated by FACS analysis after 72 h of co-culture.

### The in vivo antitumor activity of GD2/CAR-T cells in a PDX model

The PDX model was established by subcutaneously inoculating the fragments of patient-derived melanoma tissues (MMYC-3 and AMYC-5) into 6-week-old NOD/SCID (non-obese diabetic and severe combined immunodeficiency) female mice (4–6 weeks old; 18–22 g). The specific information on the construction of PDX mouse model as previously described [24].

When the tumor volume reached approximately 250 mm^3^ in approximately 30–35 days after tumor fragment inoculation, the mice were divided into five groups (4 in each group), injected with 1 × 10^7^ T cells/100 μl (GD2.BBζ CAR-T cells, non-CAR-T cells or control phosphate-buffered saline (PBS)) either systemically via the tail vein (i.v.) or locally to the tumor mass (i.t.) on days 0, 7, 14, and 21. Tumor growth was subsequently measured with calipers, and the tumor volume was calculated using the following formula: volume = length × width 2/2. When the tumor size reached approximately 2000 mm^3^, the mice were sacrificed. All animal care and experimental procedures were carried out in accordance with Animal Care Ethics and were approved by the Medical Ethics Committee of the Beijing Cancer Hospital & Institute.

### Statistical analysis

Statistical evaluation was conducted with IBM SPSS statistical software (version 20.0). The *t* test was used to analyze mean values for normally distributed continuous variables, whereas the Mann-Whitney *U* test was used to compare mean values for abnormally distributed continuous variables. The correlations between the GD2 expression status and clinical parameters were evaluated by the chi-square test or Fisher’s exact test. OS curves were estimated using the Kaplan-Meier method. Log-rank tests were used to estimate the statistical significance between the time-dependent outcomes of OS. For all statistical tests, *P* < 0.05 (two-tailed test) was considered statistically significant.

## Results

### Correlation of GD2 expression with clinicopathological features

**Fig. 1:**
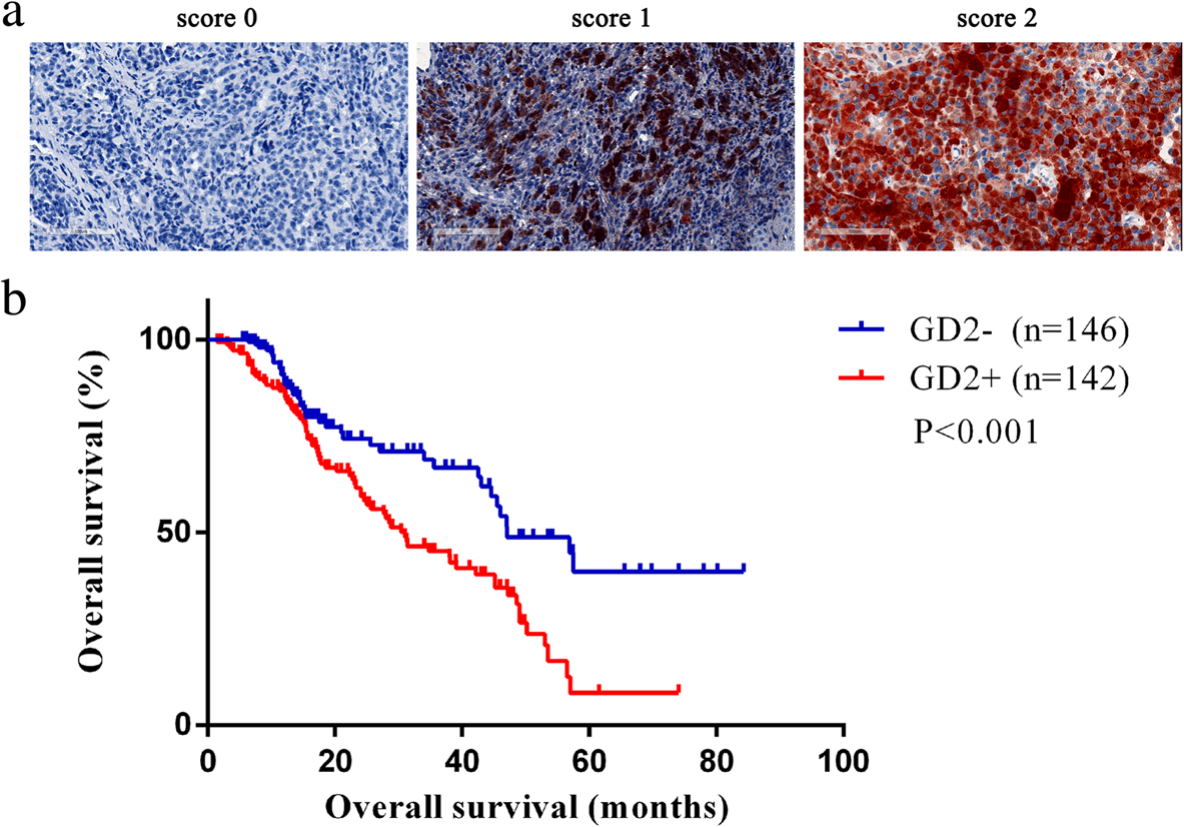
Correlation of GD2 expression with clinicopathological features. **a** Representative images of melanoma tumor cells with varying staining intensity and varying percentages of GD2-positive tumor cells (scored 0–3). A, score 0; B, score 1; and C, score 2. **b** Kaplan-Meier curves showing the correlation of GD2 expression with the overall survival of melanoma patients (*P* < 0.05)

We analyzed the expression of GD2 by immunohistochemistry, as shown in Fig. 1a. Among the 288 samples, 49.3% of the cases (142/288) demonstrated positive staining for ganglioside GD2, with a score of 1 for GD2 expression in 101 cases and a score of 2 for GD2 expression in 41 cases. The expression of ganglioside GD2 was relatively more frequent in acral (50.0%) and mucosal (56.3%) melanoma than in chronic sun-induced damage (CSD) (14.3%) and non-chronic sun-induced damage (non-CSD) (33.3%) melanoma, as shown in Table 1. We use fixed pellets of WM-266-4 (GD2^+^) cells as positive staining tissue sections and 293T(GD2^−^) cells as negative tissue sections, data shown in Additional file 2: Figure S2A, representative photomicrographs of 20 melanoma cases are shown on Additional file 2: Figure S2B.

**Table 1:** GD2 expression in melanoma

Melanoma subtypes	Number of cases	Number of cases with GD2 expression
Acral	134	67 (50.0)
Mucosal	71	40 (56.3)
CSD	14	2 (14.3)
Non-CSD	27	9 (33.3)
Unknown primary	42	24 (57.1)
Total	288	142 (49.3)

*CSD* melanomas on skin with chronic sun-induced damage, *non-CSD* melanomas on skin without chronic sun-induced damage

The median survival time in patients exhibiting ganglioside GD2 expression was significantly shorter than that in patients without ganglioside GD2 expression (31 vs. 47.1 months, *P* < 0.001, Fig. 1b). Statistically significant differences in ganglioside GD2 expression were observed between primary melanoma and metastatic melanoma (36.8 vs. 58.9%, *P* < 0.001). We also observed that GD2 expression was significantly associated with the tumor, node, and metastasis (TNM) stage (*P* < 0.05). The data are shown in Table 2.

**Table 2:** Correlation of GD2 expression to clinicopathologic factors of Chinese melanoma patients

Clinico pathologic feature	GD2+ (%)	GD2− (%)	*P* value^a^
Age (year)			
Median (range)	52.7 ± 13.5	51.1 ± 15.2	0.348
Gender *n* (%)			
Male	69 (48.6)	74 (50.7)	0.722
Female	73 (51.4)	72 (49.3)	
Ulceration *n* (%)			
Yes	82 (57.7)	84 (57.5)	0.971
No	60 (42.3)	62 (42.5)	
Thickness			
Median (range)	3.5 (0.5,20.0)	3.7 (0.5,19.5)	0.581
TNM stage *n* (%)			
I (16)	5 (3.5)	11 (7.5)	0.003
II (109)	41 (28.9)	68 (46.6)	
III (92)	55 (38.7)	37 (25.3)	
IV (71)	41 (28.9)	30 (20.5)	
Metastasis			
Yes	96 (67.6)	67 (45.9)	< 0.0001
No	46 (32.4)	79 (54.1)	
Primary site			
Acral (134)	67 (47.2)	67 (45.9)	0.017
Mucosal (71)	40 (28.2)	31 (21.2)	
CSD (14)	2 (1.4)	12 (8.2)	
Non-CSD (27)	9 (6.3)	18 (12.3)	
Unknown primary (42)	24 (16.9)	18 (12.3)	
Survival (months)			
Median (95% CI)	31 (34.1,60.1)	47.1 (21.3,40.7)	< 0.0001

^a^For evaluation of age, the two independent sample *t* test or one-way ANOVA was used. For evaluation of gender, ulceration, and TNM stage, the chi-square tests or Fisher’s exact tests were used. For evaluation of thickness, Mann-Whitney *U* tests were used. For evaluation of OS time, Log-rank tests were used

Univariate Cox analysis determined that the hazard ratios (HR) for patients with GD2 expression was 1.961 (95% CI, 1.334–2.883; *P* = 0.001). Therefore, GD2 expression together with TNM stage and metastasis may be of prognostic significance for melanoma patients, and melanoma patients with GD2 expression may be at higher risk of death, as shown in Table 3. Factors found to be significant by univariate analysis were subjected to multivariate Cox proportional hazards analysis. In the multivariate Cox analysis, TNM stage, metastasis (HR = 2.405; 95% confidence interval (CI) = 1.592–3.633, *P* < 0.0001) and GD2 expression (HR = 1.547; 95% CI = 1.032–2.317, *P* = 0.035) were identified as independent prognostic factors for OS in Chinese melanoma patients, as shown in Table 3.

**Table 3:** Cox regression analysis of GD2 expression and clinicopathologic factors with overall survival

Factors	Group	HR (95% CI)	*P* value
Univariate analysis			
Age	> 60 vs. ≤ 60	1.08 (0.736–1.585)	0.693
Gender	Male vs. female	1.005 (0.702–1.438)	0.98
Ulceration	Yes vs. no	1.218 (0.841–1.763)	0.297
Thickness	> 2 vs. ≤ 2 mm	1.526 (0.843–2.76)	0.163
TNM stage	I,II vs. III,IV	2.757 (1.852–4.105)	< 0.0001
Metastasis	Yes vs. no	2.757 (1.852–4.105)	< 0.0001
GD2 expression	GD2+ vs. GD2^−^	1.961 (1.334–2.883)	0.001
Multivariate analysis			
GD2 expression	GD2+ vs. GD2^−^	1.547 (1.032–2.317)	0.035
TNM stage (metastasis)	I,II vs. III, IV (yes vs. no)	2.405 (1.592–3.633)	< 0.0001

*CI* confidence interval

### Expression of GD2 in melanoma cell lines

**Fig. 2:**
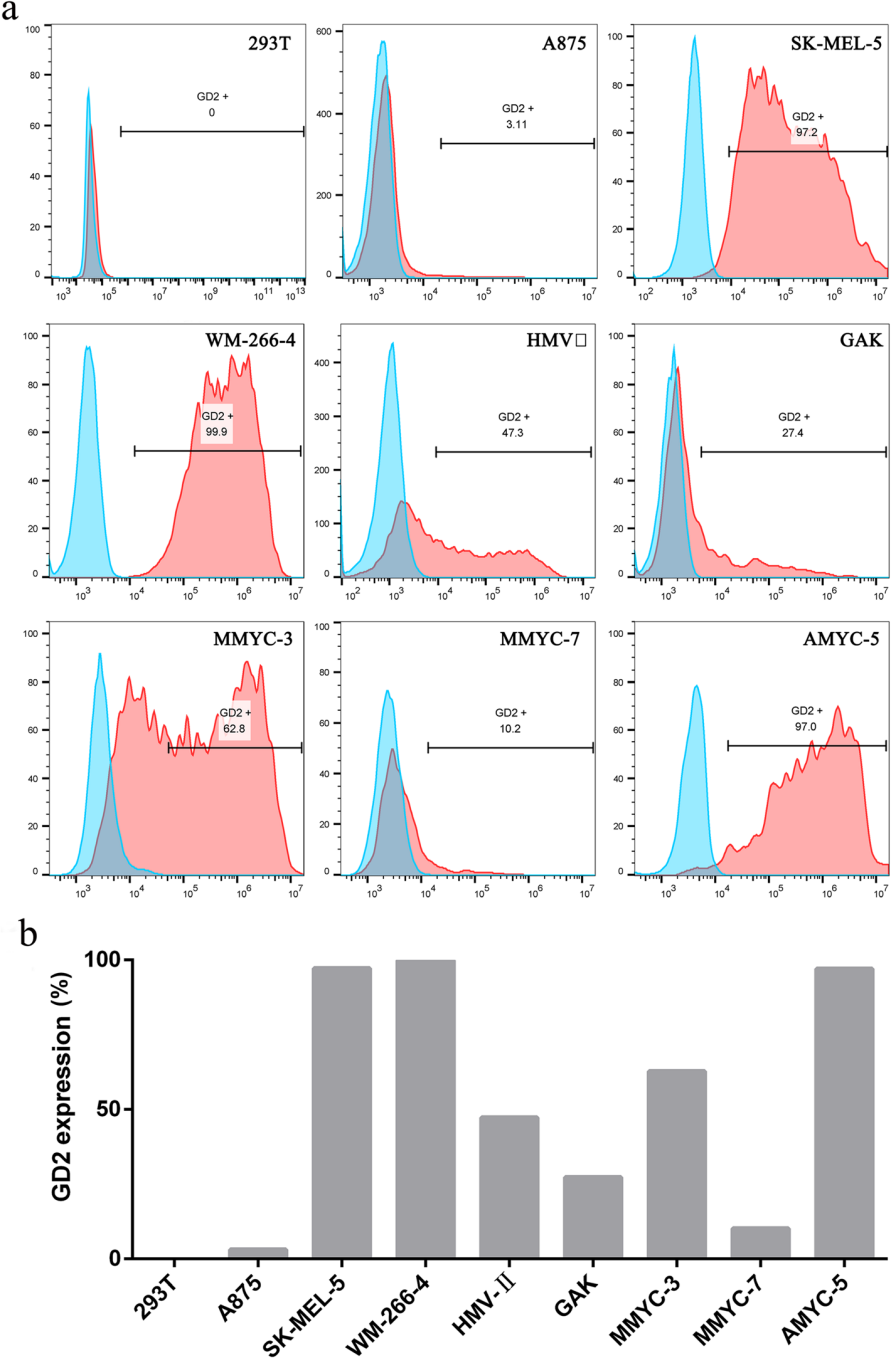
Expression of GD2 antigen in human melanoma cell lines. **a** The expression of GD2 in eight melanoma cell lines was evaluated by FACS analysis. SK-MEL-5, WM-266-4, HMV-II, GAK, MMYC-3 and AMYC-5 cell lines exhibited GD2 expression at intermediate/high (++/+++), A875 and MMYC-7 cell lines exhibited GD2 expression at low levels (+), respectively (red histograms). In 293T cell lines, GD2 was undetectable. A GD2 isotype antibody was used as a negative control for the detection of GD2 expression (blue histograms). **b** The histogram of GD2 expression in 293 T and melanoma cell lines

We utilized three primary melanoma cell lines derived from tumor tissue of the established PDX model, two mucosal melanoma cell lines, and three nonacral cutaneous melanoma cell lines in present study. GD2 expression in the melanoma cells was verified by FACS and varied between 3.1 and 99.9%, as shown in Fig. 2. Melanoma-associated chondroitin sulfate proteoglycan (MCSP) expression in the three primary melanoma cell lines was evaluated by FACS to investigate the presence of melanoma cells in the primary melanoma cell lines. All three primary melanoma cell lines expressed MCSP in various degrees, and the data are shown in Additional file 3: Figure S3.

### GD2.BBζ CAR-T cells were successfully modified by lentiviral GD2/CAR

**Fig. 3:**
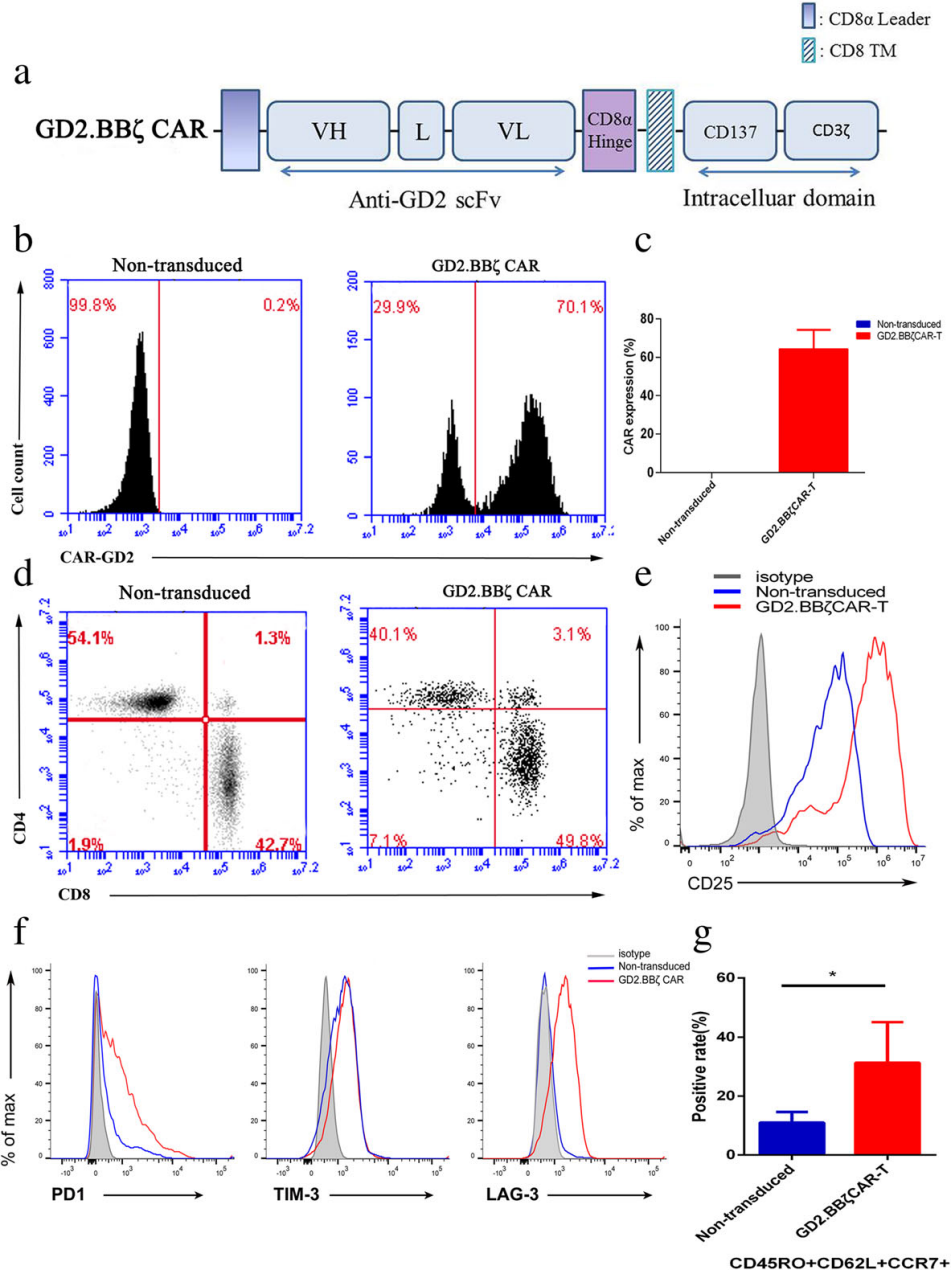
Generation of GD2/CAR-T cells in vitro. **a** Schematic representation of GD2-based CAR constructs containing the CD3 ζ cytosolic domain in combination with the CD137 costimulatory module (GD2.BBζ CAR). *VL* variable L chain, *L* linker, *VH* variable H chain, and *TM* transmembrane region. **b** The expression of CAR-GD2 was assessed by FACS analysis using the anti-idiotypic antibody 1A7 raised against anti-GD2 mAb 14G2a. The graph shows representative expression levels of CAR-GD2 in non-transduced T cells and GD2.BBζ CAR-T cells. **c** The overall transduction efficiency of CAR-T cell manufacture. **d** The expression of CAR-GD2 in CD4^+^ and CD8^+^ T lymphocytes after the gene transfer. Following the selection of GD2^+^ T cells, GD2.BBζ CAR-T cells consisted of 49.8% CD8^+^ T cells and 40.1% CD4^+^ T cells. Following the selection of GD2^−^ T cells, non-transduced T cells consisted of 54.1% CD8^+^ T cells and 42.7% CD4^+^ T cells. **e** Activation marker expression of GD2.BBζ CAR-T cells on 9 days after initial activation. **f** Exhaustion marker expression of GD2.BBζ CAR-T cells on 9 days after initial activation. **g** Tcm phenotypic features of GD2.BBζ CAR-T and non-transduced T cells were evaluated by FACS analysis on day 9 of culture initial activation. Mean positive rates ± SD from three different T cell lines are shown

The construction of lentiviral GD2/CAR is shown in Fig. 3a. After 4 days of lentiviral GD2/CAR transduction, the purity of CD3^+^ T cells was above 90% under the T cell expansion and activation culture system with CD3/CD28 and IL2. The surface expression of GD2/CAR on the T cells was confirmed by FACS using anti-idiotypic antibody 1A7 raised against anti-GD2 mAb 14G2a, as shown in Fig. 3b. GD2/CAR expression, which is representative of GD2.BBζ CAR-T cells, reached 70% among the CD3^+^ T cells. The overall transduction efficiency of CAR-T cell production is shown in Fig. 3c. After selecting for CAR^+^ T cells, the GD2.BBζ CAR-T cells consisted of 49.8% CD8^+^ T cells and 40.1% CD4^+^ T cells, as shown in Fig. 3d.

We also evaluated activation marker CD25 expression in GD2.BBζ CAR-T cells. Compared with non-transduced T cell, GD2.BBζ CAR-T cells showed high levels of overactivation 9 days after initial activation, as shown in Fig. 3e. The exhaustion markers of GD2.BBζ CAR-T cells 9 days after initial activation were lower than 30%, data shown in Fig. 3f. Only a small fraction of GD2.BBζ CAR-T cells expressed the indicated central memory T (Tcm) phenotypes 9 days after initial activation (CD45RO^+^CD62L^+^CCR7^+^, 31.33% ± 7.97%), which was significantly larger than the corresponding population of non-transduced T cells (*P* < 0.05), data shown in Fig. 3g.

### GD2.BBζ CAR-T cells demonstrated specific and efficient cytotoxicity against GD2-expressing melanoma cells

**Fig. 4:**
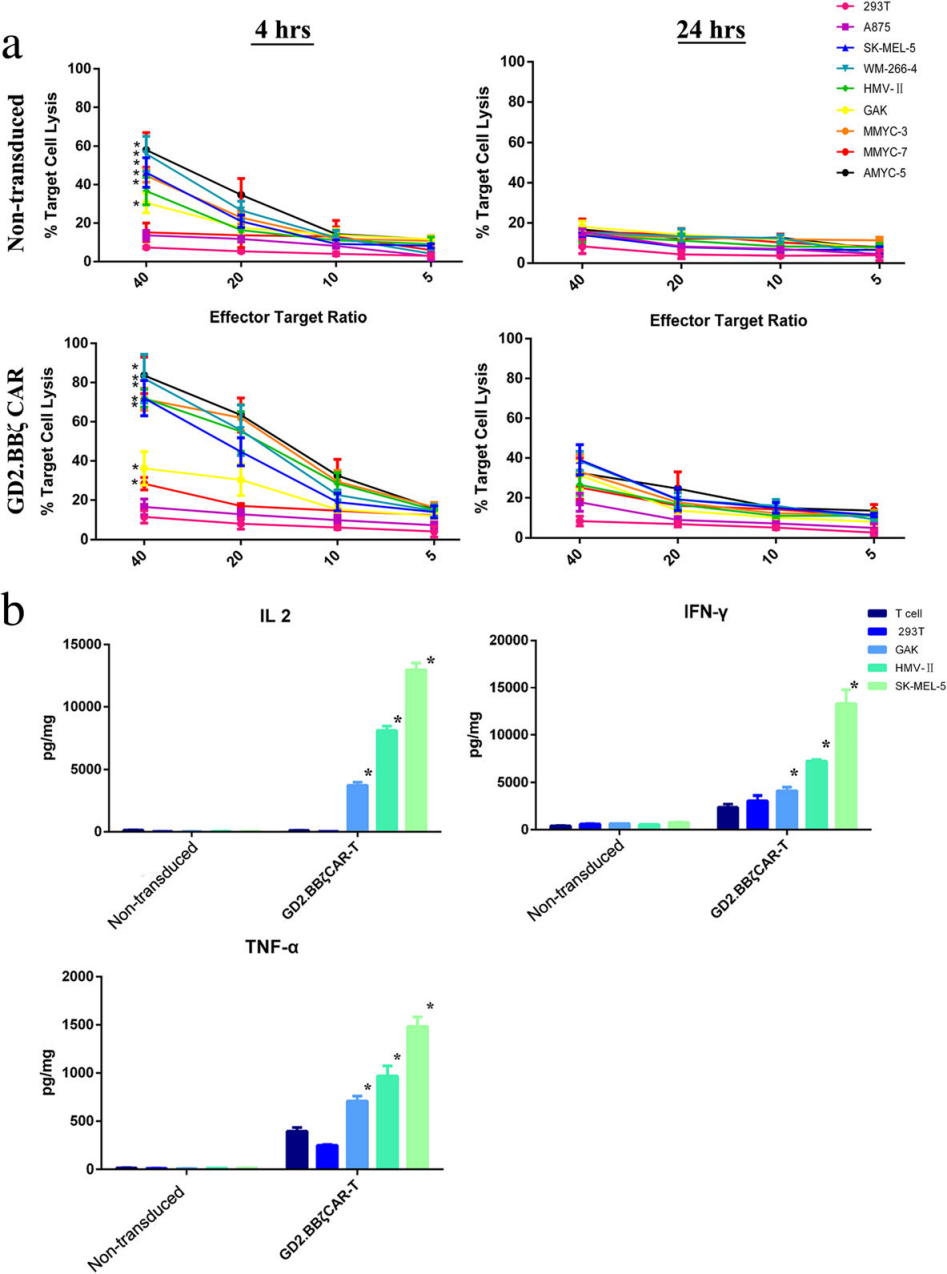
Functional analysis of GD2/CAR-T cells in vitro. **a** Cytotoxic activity of GD2/CAR-T cells. We used an LDH release assay to evaluate the cytotoxic activity of GD2.BBζ CAR-T cells and non-transduced T cells. Target cells were melanoma lines with varying GD2 expression levels. The figure illustrates the mean and SD of LDH release from 9 T cell lines after 4 h of incubation. A significant difference was detected in GD2-specific antitumor activity at each E:T ratio between GD2.BBζ CAR-T cells and control non-transduced CAR-T cells. In contrast, GD2.BBζ CAR-T cells had little antitumor activity against the GD2-negative tumor cell lines 293T, A875 (3.1% GD2 expression), and MMYC-7 (10.2% GD2 expression). **b** Th1 cytokine release of GD2/CAR-T cells. Non-transduced T cells and GD2.BBζ CAR-T cells were co-cultured (ratio of T lymphocytes:tumor cells of 20:1) with four different cell lines that were GD2-negative (293T) or were 27.4% GD2-positive (GAK), were 47.3% GD2-positive (HMV-II) or exhibited high (WM-266-4) levels of GD2-positive cells. Culture supernatant was collected 24 h later, and the production of IL-2, TNF-α, and IFN-γ were measured using a CBA assay. A substantial amount of IL2, TNF-α, and IFN-γ was released by GD2.BBζ CAR-T cells, and their releases were associated with the level of GD2 expression in the melanoma cells. In contrast, the release of IL2, TNF-α, and IFN-γ remained unchanged in non-transduced T cells or 293 T cells. The results are presented as the mean and SD from experiments that were performed in triplicate

The LDH release assay was used to determine the ability of GD2.BBζ CAR-T cells to recognize and kill melanoma cells in eight melanoma cell lines possessing varying GD2 expression levels. After 4 h of co-culture, the LDH release assays showed that the cytotoxicity of GD2.BBζ CAR-T cells correlated with the level of GD2 expression, as shown in Fig. 4a. Moreover, a robust improvement in the cytotoxicity of the GD2.BBζ CAR-T cells against the GD2-expressing melanoma cells was accompanied by an increase in the E:T ratio. Significantly different GD2 specific antitumor activity was observed at each E:T ratio between GD2.BBζ CAR-T cells and control non-transduced T cells (*P* < 0.05). In contrast, GD2.BBζ CAR-T cells had little antitumor activity against the GD2-negative tumor cell lines 293T, A875 (3.1% GD2-expressing cells) and MMYC-7 (10.2% GD2-expressing cells) (*P* < 0.05). This result demonstrates the specificity and efficiency of GD2.BBζ CAR-T cells against GD2-expressing melanoma cells.

### GD2.BBζ CAR-T cells secrete cytokines upon co-culture with GD2-expressing melanoma cells

A cytokine release assay was performed to detect whether GD2.BBζ CAR-T cells were functionally activated when co-cultured with a GD2^+^ target. A substantial amount of IL2, TNF-α, and IFN-γ was released by GD2.BBζ CAR-T cells and was associated with the quantity of GD2 expression on the melanoma cells, as shown in Fig. 4b. In contrast, the release of IL2, TNF-α, and IFN-γ remained unchanged in non-transduced T cells group or 293 T cells group. Consistent with previous research, the GD2.BBζ CAR-T cells could be activated and could exert effector cell functions in a GD2-dependent manner; moreover, 4-1BB co-stimulation enhances the production of Th1 cytokines [25]. Furthermore, we also detected the release of Th2 cytokines, include IL-4, IL-5, and IL-10. Among three cell lines group, only the GD2.BBζ CAR-T cells of WM-266-4 group release a significantly higher IL-4, IL-5, and IL-10 than control group (*P* < 0.05), as shown in Additional file 4: Figure S4.

### The activation, exhaustion, and clonal expansion characteristic of GD2.BBζ CAR-T cells in vitro

**Fig. 5:**
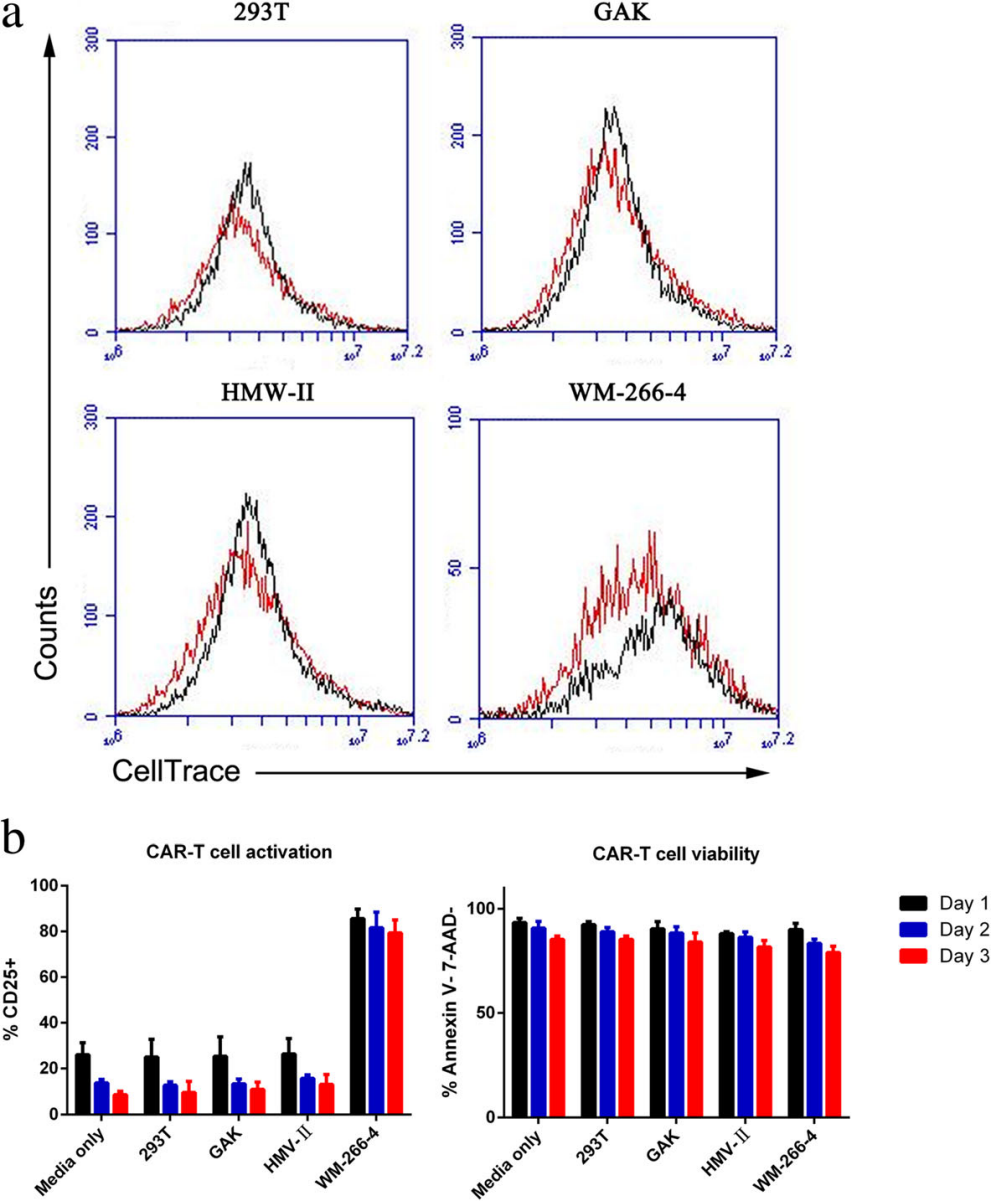
The activation, exhaustion, and clonal expansion characteristics of GD2.BBζ CAR-T cells in vitro. **a** GD2.BBζ CAR-T cells were labeled with CellTrace™ Far Red to evaluate T cell division. They were co-cultured (ratio of T lymphocytes:tumor cells of 20:1) with four different cell lines that were GD2^negative^ cell (293 T), GD2^low^ (GAK), GD2^mediate^ (HMV-II), or GD2^high^ cell (WM-266-4 cells). CellTrace™ Far Red expression in T cells was analyzed by FACS after 72 h. Only GD2.BBζ CAR-T cells underwent clonal expansion in response to GD2^+^ melanoma cell lines. Data are representative of repeat experiments. **b** “Stress test” assay was performed with GD2.BBζ CAR-T cell under repeatedly stimulation onto GD2-cell (293T), GD2+ melanoma cell (HMV-II, GAK, WM-266-4) every 24 h in vitro. The population of the CAR-T cell was evaluated by viability and activation markers (CD25), the dead cells were analyzed by Annexin V and 7-AAD staining

We investigated whether the proliferation of GD2.BBζ CAR-T cells could be stimulated by co-culture with GD2^+^ melanoma cells. As shown in Fig. 5a, GD2.BBζ CAR-T cell exhibit significant proliferation when stimulated by GD2^high^ cells (WM-266-4 cells). In contrast, when GD2.BBζ CAR-T cells were co-cultured with GD2^negative^ cells (293T), GD2^low^ cells (GAK), GD2^mediate^ cells (HMV-II) the CAR-T cell exhibited no proliferative activity.

Recent research has indicated that GD2-specific CAR-T cells undergo activation induced cell death following antigenic stimulation [26, 27]. We performed a “stress test” assay with GD2.BBζ CAR-T cells under repeated stimulation onto GD2-cells (293T), and GD2^+^ melanoma cells (HMV-II, GAK, WM-266-4) every 24 h in vitro, as shown in Fig. 5b. At each transfer, we evaluated the CAR-T cell population via viability and activation markers (CD25), and the dead cells were analyzed by Annexin V and 7-AAD staining. We did not observe significant CAR-T cell death with each stimulation.

### GD2.BBζ CAR-T cells inhibited the growth of GD2-expressing melanoma cells and exhibit persistence in PDX models

**Fig. 6:**
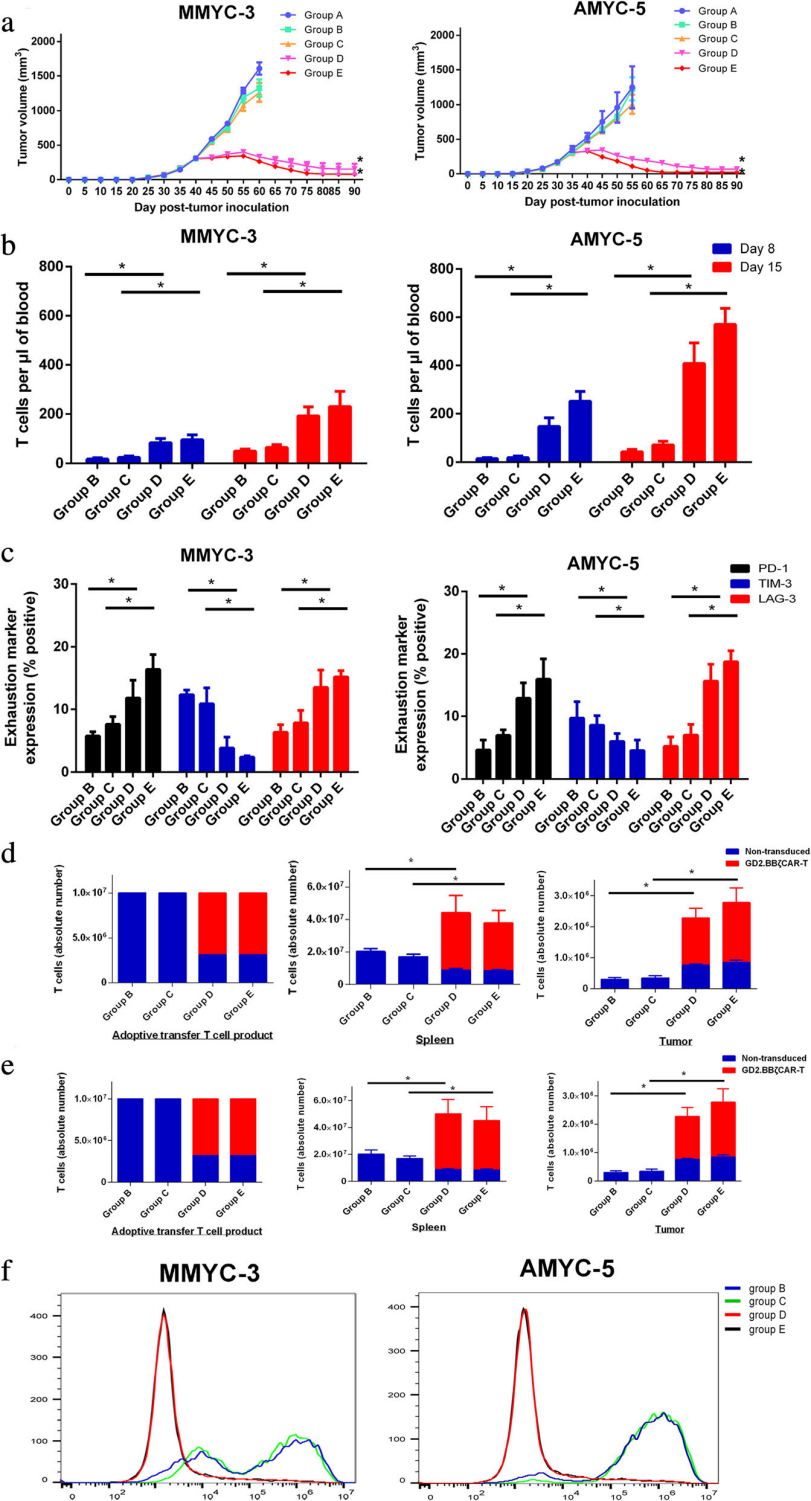
In vivo antitumor activity of GD2/CAR-T cells. **a** MMYC-3 and AMYC-5 patient-derived xenografts received different treatments: group A, PBS (i.v.); group B, non-transduced T cells (i.v.);group C, non-transduced T cells (i.t.); group D, GD2.BBζ CAR-T cells (i.v.); and group E, GD2.BBζ CAR cells (i.t.). The results are expressed as the mean tumor volume (mm3) ± SD with *n* = 5 for all groups. **b** Quantification of T cells within the blood on eight and 15 days after adoptive transfer into mice: group B, non-transduced T cells (i.v.);group C, non-transduced T cells (i.t.); group D, GD2.BBζ CAR-T cells (i.v.); and group E, GD2.BBζ CAR cells (i.t.). **c** Exhaustion marker expression of GD2.BBζ CAR-T cells on day 14 after adoptive transfer of T cells: group B, non-transduced T cells (i.v.);group C, non-transduced T cells (i.t.); group D, GD2.BBζ CAR-T cells (i.v.); and group E, GD2.BBζ CAR cells (i.t.). **d** Quantification of T cells within the spleen and tumor 15 days after adoptive transfer of T cells in MMYC-3: group B, non-transduced T cells (i.v.);group C, non-transduced T cells (i.t.); group D, GD2.BBζ CAR-T cells (i.v.); and group E, GD2.BBζ CAR cells (i.t.). **e** Quantification of T cells within the spleen and tumor 15 days after adoptive transfer of T cells in AMYC-5: group B, non-transduced T cells (i.v.); group C, non-transduced T cells (i.t.); group D, GD2.BBζ CAR-T cells (i.v.); and group E, GD2.BBζ CAR cells (i.t.). **f** The GD2 expression of tumors on 15 days after adoptive transfer of 10^7^ T cells: group B, non-transduced T cells (i.v.); group C, non-transduced T cells (i.t.); group D, GD2.BBζ CAR-T cells (i.v.); and group E, GD2.BBζ CAR cells (i.t.). *P < 0.05 by Student’s *t* test

We developed a PDX model by inoculating MMYC-3 and AMYC-5 tissue into NOD/SCID mice. The tumor volume of mice treated with PBS or non-transduced T cells increased rapidly. In contrast, mice receiving i.v. or i.t. injections of GD2.BBζ CAR-T cells experienced rapid tumor regression, which confirmed the potent antitumor activity of GD2.BBζ CAR-T cells in vivo (*P* < 0.05), as shown in Fig. 6a. The representative image of the tumor is shown in Additional file 5: Figure S5. The cytotoxic efficacy of GD2.BBζ CAR-T cells when delivered via i.t. injection was higher than that when delivered via i.v. injection.

Recent clinical trials have shown that compared with those incorporating CD28 co-stimulatory domains, CD19 CAR-T cells incorporating 4-1BB co-stimulatory domains exhibit prolonged persistence [28, 29]. We sought to evaluate the persistence of CAR-T cells. After adoptive transfer of 10^7^ T cells 8 and 15 days, the quantification of GD2.BBζ CAR-T cells was higher than that of non-transduced T cell in vivo, as shown in Fig. 6b. Exhausted T cells demonstrate limited persistence in vivo, so we evaluated the expression of exhaustion marker of GD2.BBζ CAR-T cells 15 days after adoptive transfer of 10^7^ T cells, the expression of PD-1, LAG-3, and TIM-3 was lower than 25%, as shown in Fig. 6c. We also measured the quantification of T cells within the spleens and tumors 15 days after adoptive transfer of 10^7^ T cells, which was significantly higher in the GD2.BBζ CAR-T cells groups than in the non-transduced group, as shown in Fig. 6d, e. The GD2 expression of tumors on 15 days after adoptive transfer of 10^7^ T cells was evaluated by FACS. Compared with control group, the GD2 expression of the tumor in GD2.BBζ CAR-T cells group declined to blow 10%, as shown in Fig. 6f.

## Discussion

Melanoma is a highly aggressive skin cancer, and several immune-related therapies have been approved by the US FDA for its treatment, including interleukin 2 (IL-2), interferon-α (IFN-α), cytotoxic T cell-stimulating cytokine (CTLA-4) and programmed cell death protein 1 (PD-1) blocking antibodies [30], all of which target T cell activation. Adoptive cell therapy (ACT) is an alternative immunotherapy for melanoma, and its primary mechanism is T cell activation. While earlier clinical research mainly focused on ACT for tumor-infiltrating lymphocytes (TILs) [31], efforts have recently been diverted to the generation of T cells with T cell receptors (TCRs) specific for tumor-associated antigens (TAAs), including NY-ESO-1 [32] and MART-1 [33]. The TCRs recognize antigens presented by MHC molecules, which limit the number of patients eligible for this immunotherapy, whereas CAR-T cells are activated upon recognizing unprocessed structures on the surface of the target in an MHC-independent manner [34]. Due to advantages in stable antigen identification, the reduction of immunosuppressive tumor microenvironments and treatment toxicities, and the prevention of antigen escape, CAR-T cells have been widely explored and applied as a cancer therapy [35, 36].

GD2 is overexpressed in melanoma, neuroblastoma, and small-cell lung cancer, but its expression is limited in normal tissues [37]; therefore, targeting GD2 could reduce the incidence rate of toxicity associated with off-target or on-target/off-target effects. In our study, the rate of GD2 expression is 49.3% among 288 cases. The expression of ganglioside GD2 is more frequent in acral and mucosal melanoma than in CSD and non-CSD subtypes, which are the major melanoma subtypes in Caucasian cohorts [38]. Due to the small sample size of CSD and non-CSD melanoma subtypes, statistical biases may be produced. The higher expression of ganglioside GD2 in metastatic melanoma compared to primary melanoma was consistent with the notion that GD2 expression is related to increased metastatic potential [39]. Moreover, our study demonstrates that the expression of GD2 is significantly associated with poor prognosis. Hence, GD2 is an attractive target for CAR-T therapy.

Clinical trials of anti-GD2 monoclonal antibodies have demonstrated that the agent could significantly improve the survival of neuroblastoma patients [40], but for melanoma patients, the benefits are limited due to the varying GD2 expression levels in melanoma (usually lower), which influence the capacity to bind with anti-GD2 monoclonal antibodies. However, several studies have indicated the CAR-T cells could induce complete cytotoxic responses to tumor cells despite the low expression of target antigen [18, 41]. Our study also shows that GD2.BBζ CAR-T cells could lyse GD2+ melanoma (27.4–99.9% GD2 expression), including melanoma cells with low GD2 expression. Antibodies possessing multiple antibody-derived binding domains on their cell surface exhibited improved cytotoxic ability compared to bivalent antibodies in solution, and thus, CAR-T cells exhibit superior cytolytic activity compared to antibodies [42].

Similar with most malignancies, melanoma cells lack the expression of T cell costimulatory molecules, which could trigger the complete activation of T lymphocytes against TAAs via their native or chimeric receptors. To activate the effector function and prolong the persistence of T cells, we introduced the CD137 (4-1BB) costimulatory signaling domain into the GD2 chimeric receptor. CD137 belongs to the TNF receptor family, which is essential for the proliferation and survival of T cells, particularly for memory T cell responses [43, 44]. A previous study indicated that the adoptive transfer of tumor-specific T cells co-stimulated ex vivo with 4-1BBL exhibited increased persistence and antitumor activity in vivo [45]. Our study also demonstrated that GD2.BBζ CAR-T cells could undergo clonal expansion when co-cultured with GD2^+^ melanoma cell lines. T cell exhaustion is a major factor restricting the efficacy of CAR-T therapies, and 4-1BB could ameliorate exhaustion by reducing the expression of known exhaustion-related genes and by modulating metabolic, apoptosis, and hypoxia pathways [22]. Several clinical trials have demonstrated that CAR-T cells harboring the 4-1BB costimulatory domain exhibit longer persistence than those harboring the CD28 costimulatory domain [46–48]. Moreover, the 4-1BB costimulatory signaling domain could endow T cells with superior proliferative potential, more potent antitumor activity and a Th1-based cytokine profile [25]. In the present study, we show that GD2.BBζ CAR-T cells could preferentially secrete high levels of Th1 cytokines, including IL-2, TNF-α, and IFN-γ, upon encountering a tumor cell and exert strong antitumor activity in vitro. Moreover, the GD2.BBζ CAR-T cells exhibit persistence in vivo.

In our study, we investigate the performance of CAR-T therapy using CD3^+^ T cells instead of purified CD8^+^ cytotoxic lymphocytes (CTLs) because CD4^+^ T cells have been confirmed to increase the function of CD8^+^ T cells [49]. The results of our PDX model experiments demonstrate that CD3^+^ T cells transduced with GD2.BBζ CAR show higher cytotoxicity than the non-transduced T cells, which consists with previous research findings that adoptive transfer of mixed populations of antigen-specific CD8^+^ T cells and CD4^+^ T cells promotes overall antitumor immunity. With regard to the administration route of T cells, both locally intratumor injection and venous injection lead to tumor regression, which confirms the capacity of CAR-T cells to circulate, traffic to the tumor, and perform cytotoxic ability. Although venous injections are favorable in clinical applications due to the ease of administration and the efficacy displayed in the preclinical model, several preclinical, and clinical studies have demonstrated the effectiveness of locally injected CAR-T cells [49–51]. Our study suggests that local delivery of T cells for solid tumor may lead to a promising therapeutic efficacy, which may partly due to increased transmission of T cells to the tumor and to provide a favorable E/T ratio. However, local delivery of T cells may not be suitable for tumors with multiple metastatic.

There are some limitations and potential perspectives in our study. In present study, the GD2.BBζ CAR-T cells could expand when stimulated by GD2^high^ cell (WM-266-4 cells), whereas fail to expand in response to GD2^low^ (GAK), GD2^mediate^ (HMV-II). In previous study, the GPC3-4-1BB-CAR-T cells exhibit proliferation when stimulated by GPC3-positive cell in vitro [52]. Moreover, the GD2-CD28-OX40-CAR-T cells could expand when stimulated by GD2-positive cell [53]. The association between the proliferation of CAR-T cell and quantity of GD2 expression on the co-cultured target cells remain unknown, which need further study to verify.

## Conclusions

Our study demonstrated that GD2.BBζ CAR-T cells can efficiently lyse melanoma in a GD2-specific manner and release Th1 cytokines in an antigen-dependent manner. Anti-GD2/4-1BB CAR-T cells represent a clinically appealing treatment strategy for Chinese melanoma patients with GD2 expression, thus providing a basis for additional studies in the clinical application of immunotherapy for melanoma.

## Additional files

Additional file 1: Figure S1. Structure of the viral vector. (TIFF 3119 kb)

Additional file 2: Figure S2. (A) Staining of WM-266-4 (GD2+) and 293T(GD2-). (B) Representative photomicrograph of 20 melanoma cases. (TIFF 21902 kb)

Additional file 3: Figure S3. Expression of MCSP in primary melanoma cell lines. (TIFF 1807 kb)

Additional file 4: Figure S4. Th2 cytokine release of GD2/CAR-T cells. Non-transduced T cells and GD2.BBζ CAR-T cells were co-cultured (ratio of T lymphocytes:tumor cells of 20:1) with four different cell lines that were GD2-negative (293T) or were 27.4% GD2-positive (GAK) and were 47.3% GD2-positive (HMV-II) or exhibited high (WM-266-4) levels of GD2-positive cells. Culture supernatant was collected 24 h later, and the production of IL-4, IL-5, and IL-10 were measured using a CBA assay. The results are presented as the mean and SD from experiments that were performed in triplicate. *P < 0.05 by Student’s *t* test. (TIFF 412 kb)

Additional file 5: Figure S5. Image of a representative tumor in the PDX models in which GD2.BBζ CAR-T cells inhibited the growth of GD2-expressing melanoma cells. Group A, PBS (i.v.); group B, non-transduced T cells (i.v.); group C, non-transduced T cells (i.t.); group D, GD2.BBζ CAR-T cells (i.v.); and group E, GD2.BBζ CAR cells (i.t.). (TIFF 8545 kb)

## Abbreviations

ACT: Adoptive cell therapy
AEC: 3-Amino-9-ethylcarbazole
AJCC: American joint committee on cancer
ATCC: American Type Culture Collection
CAR: Chimeric antigen receptor
CBA: Cytometric bead array
CR: Complete response
CSD: Chronic sun-induced damage
CTLA-4: Cytotoxic T cell-stimulating cytokine
CTLs: CD8^+^ cytotoxic lymphocytes
DMEM: Dulbecco’s Modified Eagle Medium
FACS: Fluorescence-activated cell sorting
FDA: Food Drug and Administration
FFPE: Formalin-fixed, paraffin-embedded
HR: Hazard ratios
IFN-α: Interferon-α
IFN-γ: Interferon-γ
IHC: Immunohistochemistry
IL-10: Interleukin-10
IL-2: Interleukin-2
IL-4: Interleukin-4
IL-5: Interleukin-5
MCSP: Melanoma-associated chondroitin sulfate proteoglycan
MHC: Major histocompatibility complex
MoAb: Monoclonal antibody
non-CSD: Non-chronic sun-induced damage
OS: Overall survival
PD-1: Programmed cell death protein 1
PDX: Patient-derived xenograft
SD: Standard deviation
TAAs: Specific for tumor-associated antigens
TCRs: T cell receptors
TILs: Tumor-infiltrating lymphocytes
TM: Transmembrane
TNF-α: Tumor necrosis factor
TNM: Tumor, node, and metastasis
