# Supplementary figures and images for: Anti-GD2/4-1BB chimeric antigen receptor T cell therapy for the treatment of Chinese melanoma patients
Source: J Hematol Oncol. 2018 Jan 3;11:1. doi: 10.1186/s13045-017-0548-2 (PMC5751546; doi:10.1186/s13045-017-0548-2)

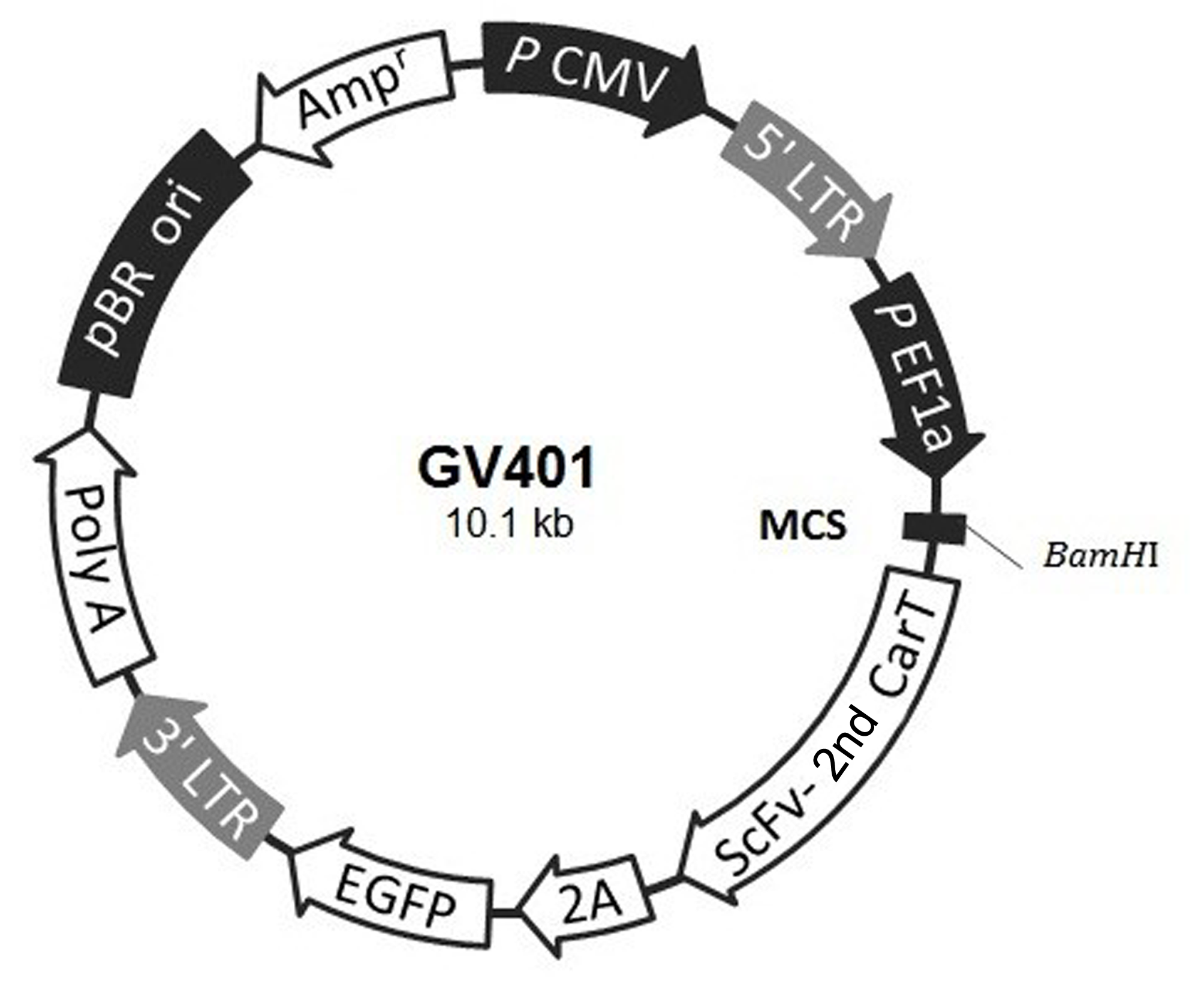

Supplement: Supplementary file 1 — Structure of the viral vector. (TIFF 3119 kb) [file 13045_2017_548_MOESM1_ESM.tif]

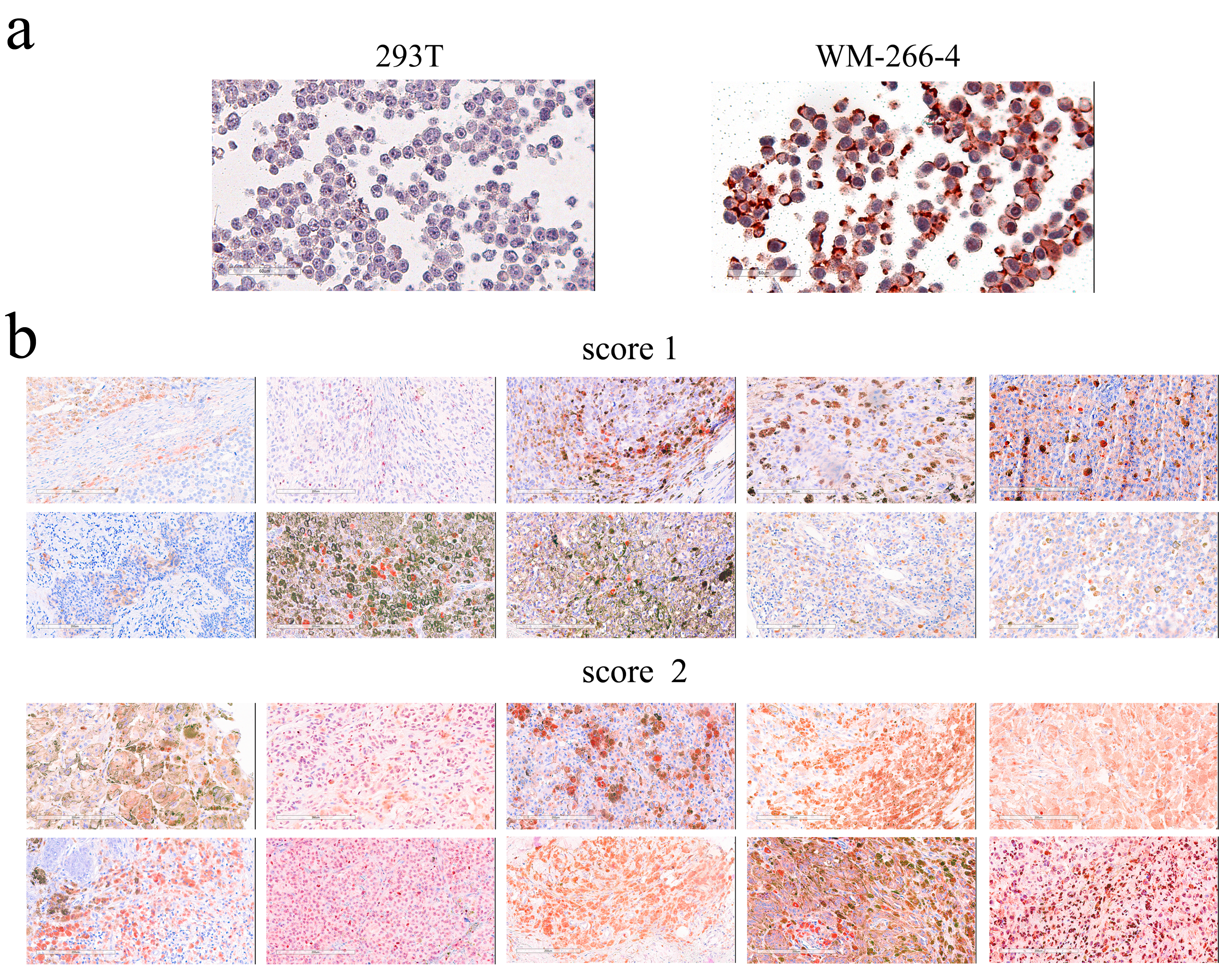

Supplement: Supplementary file 2 — (A) Staining of WM-266-4 (GD2+) and 293T(GD2-). (B) Representative photomicrograph of 20 melanoma cases. (TIFF 21902 kb) [file 13045_2017_548_MOESM2_ESM.tif]

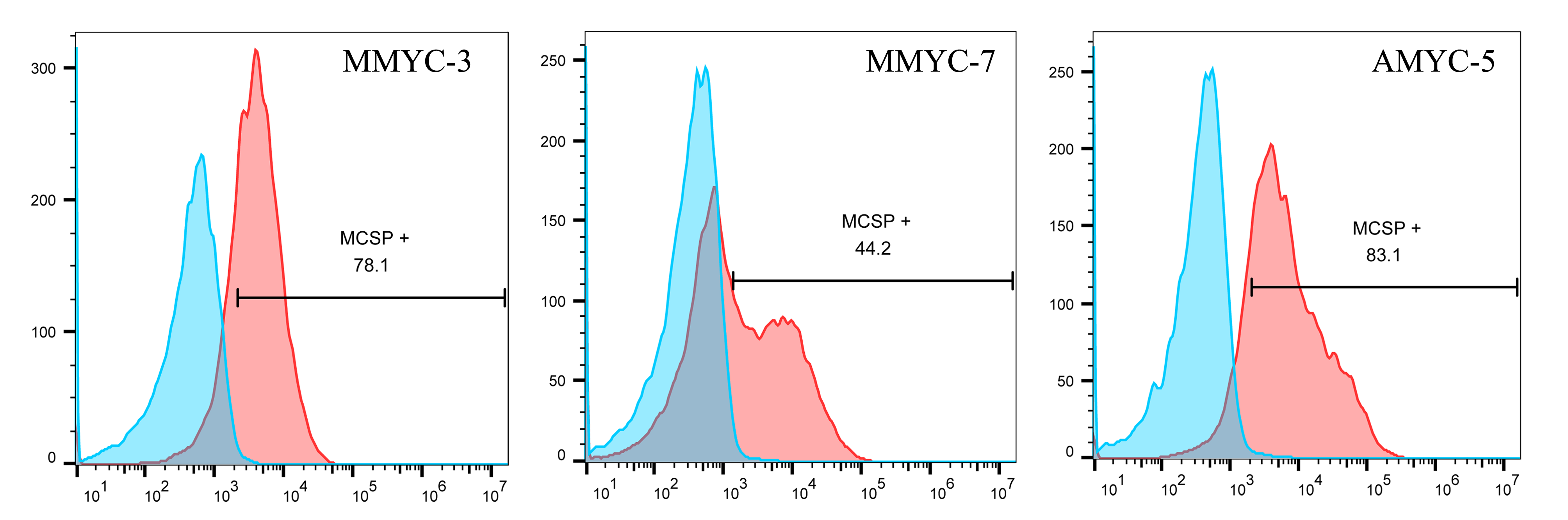

Supplement: Supplementary file 3 — Expression of MCSP in primary melanoma cell lines. (TIFF 1807 kb) [file 13045_2017_548_MOESM3_ESM.tif]

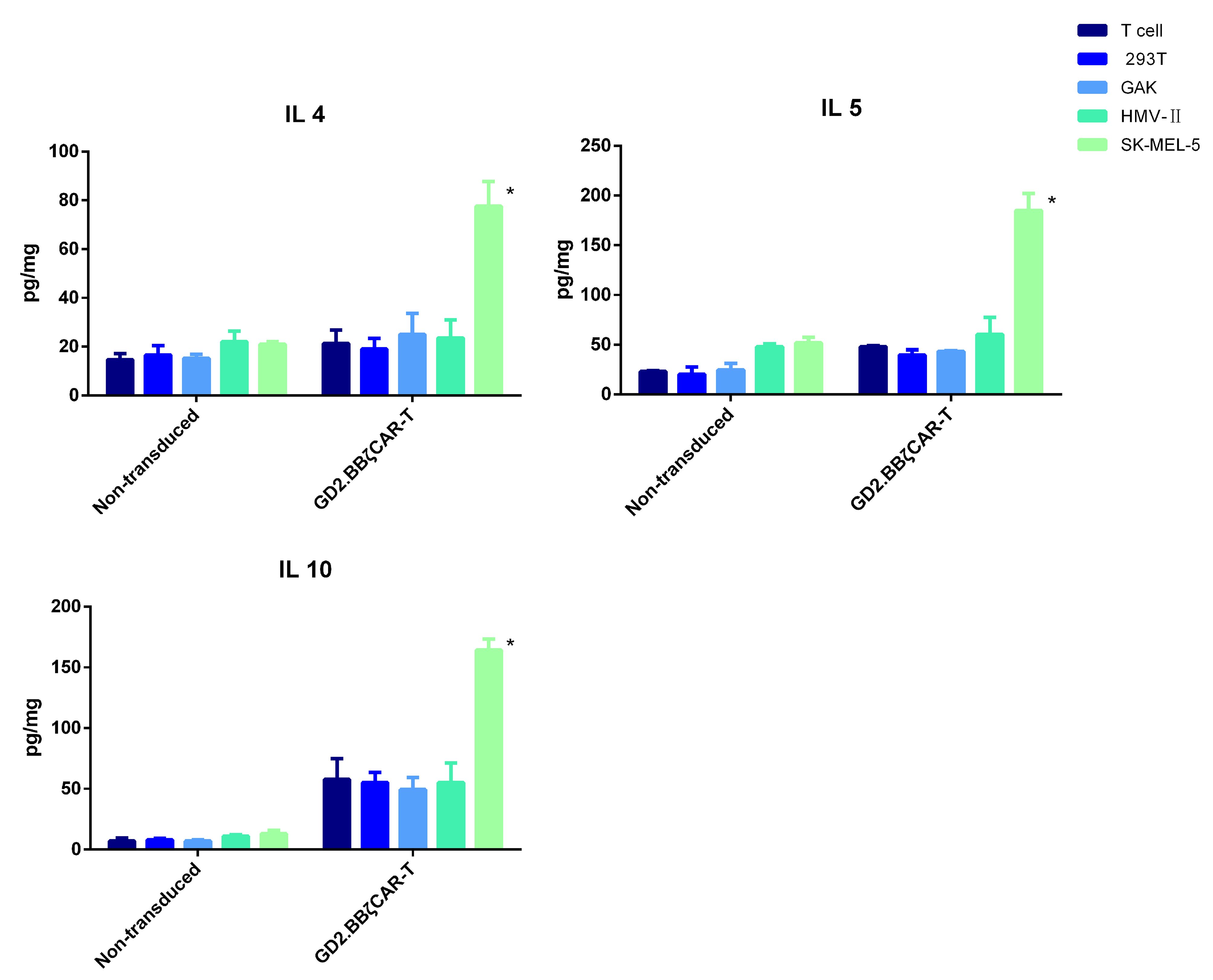

Supplement: Supplementary file 4 — Th2 cytokine release of GD2/CAR-T cells. Non-transduced T cells and GD2.BBζ CAR-T cells were co-cultured (ratio of T lymphocytes:tumor cells of 20:1) with four different cell lines that were GD2-negative (293T) or were 27.4% GD2-positive (GAK) and were 47.3% GD2-positive (HMV-II) or exhibited high (WM-266-4) levels of GD2-positive cells. Culture supernatant was collected 24 h later, and the production of IL-4, IL-5, and IL-10 were measured using a CBA assay. The results are presented as the mean and SD from experiments that were performed in triplicate. *P < 0.05 by Student’s t test. (TIFF 412 kb) [file 13045_2017_548_MOESM4_ESM.tif]

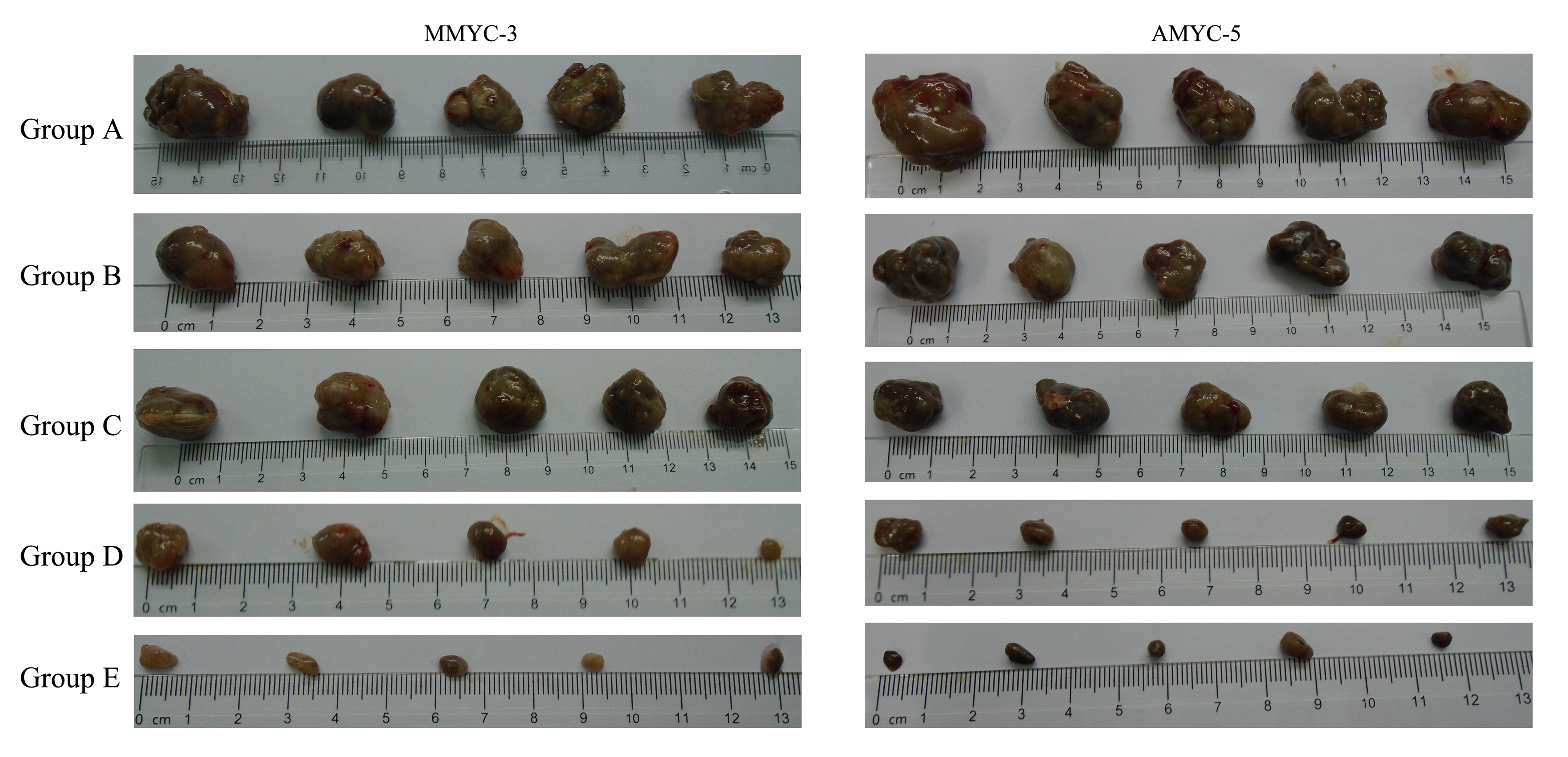

Supplement: Supplementary file 5 — Image of a representative tumor in the PDX models in which GD2.BBζ CAR-T cells inhibited the growth of GD2-expressing melanoma cells. Group A, PBS (i.v.); group B, non-transduced T cells (i.v.); group C, non-transduced T cells (i.t.); group D, GD2.BBζ CAR-T cells (i.v.); and group E, GD2.BBζ CAR cells (i.t.). (TIFF 8545 kb) [file 13045_2017_548_MOESM5_ESM.tif]
